# Spectroscopic characterization and crystal structures of two cathinone derivatives: *N*-ethyl-2-amino-1-phenylpropan-1-one (ethcathinone) hydrochloride and *N*-ethyl-2-amino-1-(4-chlorophenyl)propan-1-one (4-CEC) hydrochloride

**DOI:** 10.1007/s11419-016-0345-6

**Published:** 2016-10-31

**Authors:** Piotr Kuś, Joachim Kusz, Maria Książek, Ewelina Pieprzyca, Marcin Rojkiewicz

**Affiliations:** 1 Department of Chemistry, University of Silesia, 9 Szkolna Street, 40-006 Katowice, Poland; 2 Institute of Physics, University of Silesia, 4 Uniwersytecka Street, 40-007 Katowice, Poland; 3 Department of Forensic Medicine, Medical University of Silesia, 15 Poniatowskiego Street, 40-055 Katowice, Poland

**Keywords:** Ethcathinone, 4-Chlorocathinone, 4-CEC, NMR, MS, X-ray crystallography

## Abstract

Comprehensive chemical characterization for two cathinone derivatives, *N*-ethyl-2-amino-1-phenylpropan-1-one (ethcathinone) hydrochloride and *N*-ethyl-2-amino-1-(4-chlorophenyl)-propan-1-one (4-chloroethcathinone, 4-CEC) hydrochloride, in material seized by drug enforcement agencies was performed by nuclear magnetic resonance (NMR) spectroscopy, infrared spectroscopy, gas chromatography–mass spectrometry in positive electron ionization mode, liquid chromatography–mass spectrometry in positive electrospray ionization mode and X-ray crystallography. The examined samples of these two compounds proved to be very pure for ethcathinone and mixed with very small quantities of other substances for 4-CEC by NMR spectroscopy and mass spectrometry. X-ray crystallographic studies confirmed the occurrence of both compounds as racemic mixtures. These spectroscopic and crystallographic data seem very useful for their identification. Especially for 4-CEC, this is the first description on its spectroscopic characterization in a scientific context to our knowledge.

## Introduction

Synthetic cathinones are based on naturally occurring cathinone, one of the psychoactive compounds present in khat (*Catha edulis*), a plant growing in the Arabian Peninsula, Ethiopia and Somalia, as well as cultivated in other East African countries. The plant is variably called Arabian Tea, Abyssinian Tea, Chat Tree, Khat or Cafta. Its leaves contain ca. 1% alkaloids. The main ones are: cathinone (2-amino-1-phenylopropan-1-one, α-aminopropiophenone), l-ephedrine and cathin [(1s, 2s)-2-amino-1-phenylpropan-1-ol, (+)-nor-ψ-ephedrine].

Synthetic cathinones first appeared on the European illicit drug market in 2005 and today they make up one of the largest categories of novel psychoactive substances (NPSs) as over 80 synthetic cathinone derivatives were detected via the EU Early Warning System between 2005 and 2014 [1]. Cathinone derivatives are claimed to have effects similar to those of cocaine, amphetamine or MDMA (ecstasy), but little is known about their toxicity and pharmacokinetic properties. Synthetic cathinones most often are sold as powders or tablets through physical retail shops or via the Internet labeled as ‘plant feeders’, ‘plant food’, ‘research chemical’, or ‘bath salts’. Various products are labeled with such warnings as ‘not for human consumption' or ‘not tested for hazards or toxicity'. The availability of high-quality and pure synthetic cathinones has sometimes been reported as offering direct competition to low-quality and relatively more expensive established drugs. The most common administration routes include insufflation (snorting) and oral ingestion of capsules, tablets or powder wrapped in a cigarette paper (so-called ‘bombing’). Rectal insertion and intravenous, subcutaneous and intramuscular injections were also reported [2–4].

Ethcathinone (compound **1**) was one of the most common NPSs on the illegal drug market in Poland between 2012 and 2014 [5, 6]. In Poland, since July 2015, ethcathinone has the status of an controlled substance. Compound **1** (ethcathinone) was subjected to pharmacological studies because it is a metabolite of diethylpropion (*N*,*N*-diethyl-2-amino-1-phenylpropan-1-one), which proved to be more potent than its parent compound. This led to the conclusion that it is the metabolite, and not the starting diethylpropion, that causes the specific anorectic effect [7]. Compound **2** (4-chloroethcathinone, 4-CEC) had been briefly studied in terms of its potential anorectic effect [8]. Its current availability on the illegal drug market makes it probable that this compound will be taken off the register of medical drug candidates.

Only the ^1^H nuclear magnetic resonance (NMR) [7] and Fourier transform infrared (FTIR) [9] spectra of compound **1** have been published. Also a few spectroscopic data for compound **2** are available on the Internet only [10]. Both compounds are similar to another synthetic cathinone used recreationally and described earlier, i.e., *N*-ethyl-2-amino-1-(4-methylphenyl)propan-1-one hydrochloride (short name: 4-MEC) [11]. Compound **1** was examined chromatographically [9] and some mass spectrometry (MS) data were reported for this cathinone [9, 12, 13].

**Fig. 1:**
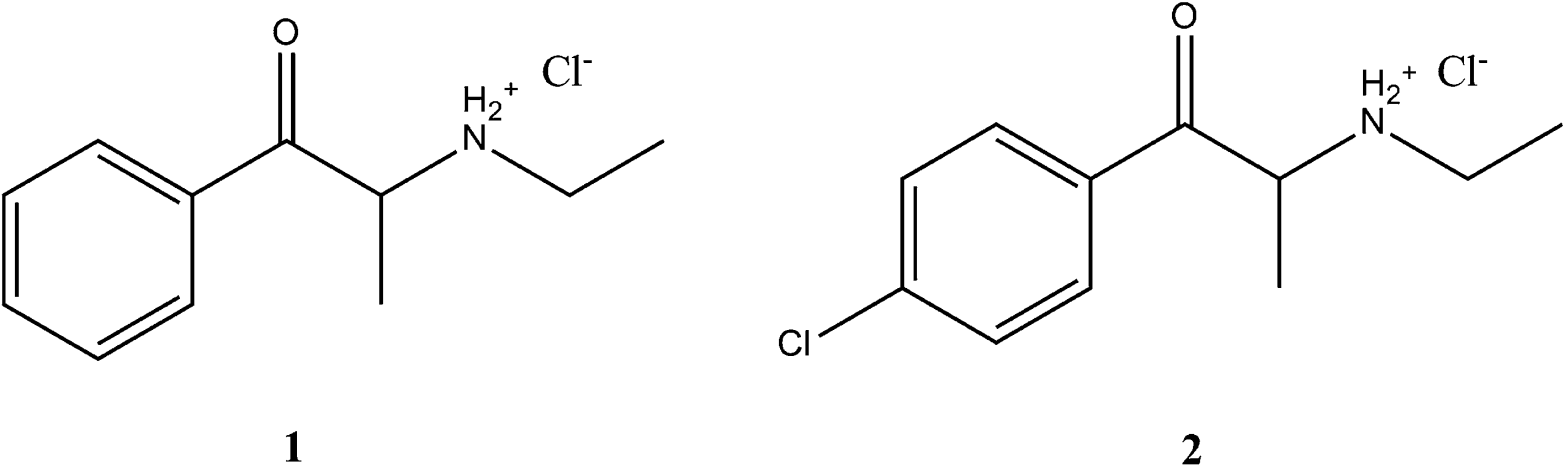
Structures of *N*-ethyl-2-amino-1-phenylpropan-1-one (ethcathinone) hydrochloride (compound **1**) and *N*-ethyl-2-amino-1-(4-chlorophenyl)propan-1-one (4-CEC) hydrochloride (compound **2**)

Both compounds can be synthesized [12, 14, 15] starting from suitable racemic mixtures of 2-bromopropiophenones with ethylamine. The products of this reaction are largely racemic mixtures of ethcathinone hydrochloride (compound **1**; in case of using 2-bromopropiophenone) or 4-chloroethcathinone (4-CEC) hydrochloride (compound **2**; in case of using 4′-chloro-2-bromopropiophenone) (Fig. 1).

Samples of the compounds **1** and **2** analyzed here by spectroscopy and crystallography exclusively originated from material seized by drug enforcement agencies. They either did not contain additional substances (in case of compound **1**), or were mixed with small quantities of other substances which allowed, however, mechanical separation of thick crystals (compound **2)** of very good purity.

In this study, we report the spectroscopic characteristics and crystallographic structures of compounds **1** and **2**. To our knowledge, such data are unavailable, especially for compound **2**.

## Materials and methods

Compounds **1** and **2** were provided by drug enforcement agencies either in pure form (compound **1**), or as a mixture of crystals (compound **2**) with less than 1% of ingredients (according to NMR data). Crystals of compound **1** suitable for crystallography were obtained by very slow evaporation of solutions used to study this compound by NMR spectroscopy. The pure compound **2** crystals were mechanically separated, recrystallized from a mixture of dichloromethane and acetone, and used in this study.

^1^H NMR spectra were recorded in D_2_O or dimethyl sulfoxide (DMSO)-*d*_6_ using a Varian spectrometer (400 MHz) (Varian, Palo Alto, CA, USA). The peaks were referenced to the residual H_2_O (4.63 ppm) and DMSO (2.49 and 39.5 ppm) resonances in ^1^H and ^13^C NMR. Infrared (IR) spectra were recorded on a Bio-Rad FTS-600 (Bio-Rad Laboratories, Hercules, CA, USA) with the samples in the form of KBr pellets. Liquid chromatography–mass spectrometry (LC–MS) analysis of samples was performed on a Thermo Scientific TSQ Quantum Access Max LC–MS operating in positive electrospray ionization (ESI) mode (Thermo Scientific, Waltham, MA, USA). Separation was achieved on a BDS Hypersil C18 (150 × 2.1 mm, 5 μm) column (Thermo Scientific) maintained at 25 °C. The mobile phase A was water, which contained 0.2% formic acid and 2 mM of ammonium formate, and phase B was acetonitrile with 0.2% formic acid and 2 mM of ammonium formate. The gradient program was applied. The operational parameters of the ESI source were as follows: vaporizing temperature 350 °C; pressure of the nebulizing gas 40 psi; capillary potential 3500 V. Gas chromatography–mass spectrometry (GC–MS) analyses were performed using a gas chromatograph (TRACE 1300 gas chromatograph) coupled to a mass spectrometer (ISQ LT) equipped with a quadruple mass analyzer (Thermo Scientific). The injector was maintained at 280 °C. Sample injection was in splitless mode. Sample component separation was conducted on an RTX-5 capillary column (30-m length, 0.25-mm inner diameter, 0.25-µm film thickness; Restek, Bellefonte, PA, USA). Helium was used as a carrier gas at the flow rate of 1.0 mL/min. The temperature program consisted of three segments: the initial column temperature (75 °C) was maintained for 1 min, then was increased linearly at 25 °C/min up to 280 °C, and maintained for 20.8 min. The mass detector was set to positive electron ionization (EI) mode, and the electron beam energy was 70 eV. The mass detector was operating in a full scan mode in the range of *m/z* 40–450.

The single crystal X-ray experiments were performed at room temperature. The data were collected using a SuperNova kappa diffractometer with an Atlas charge coupled device (CCD) detector and a Xcalibur diffractometer with a Sapphire3 CCD detector (Oxford Diffraction Ltd., Yarnton, England). For the integration of the collected data, the CrysAlis RED software (version 1.171.32.29; Agilent Technologies, Santa Clara, CA, USA) was used. The solving and refining procedures were similar for both compounds. The structures were solved using direct methods with the SHELXS97 software and the solutions were refined using the SHELXL-2014/7 program [16]. CCDC 1481895 (**1**) and 1481896 (**2**) contain supplementary crystallographic data for this paper. These data can be obtained free of charge from The Cambridge Crystallographic Data Centre via: www.ccdc.cam.ac.uk/data_request/cif.

## Results and discussion

### NMR spectra

**Fig. 2:**
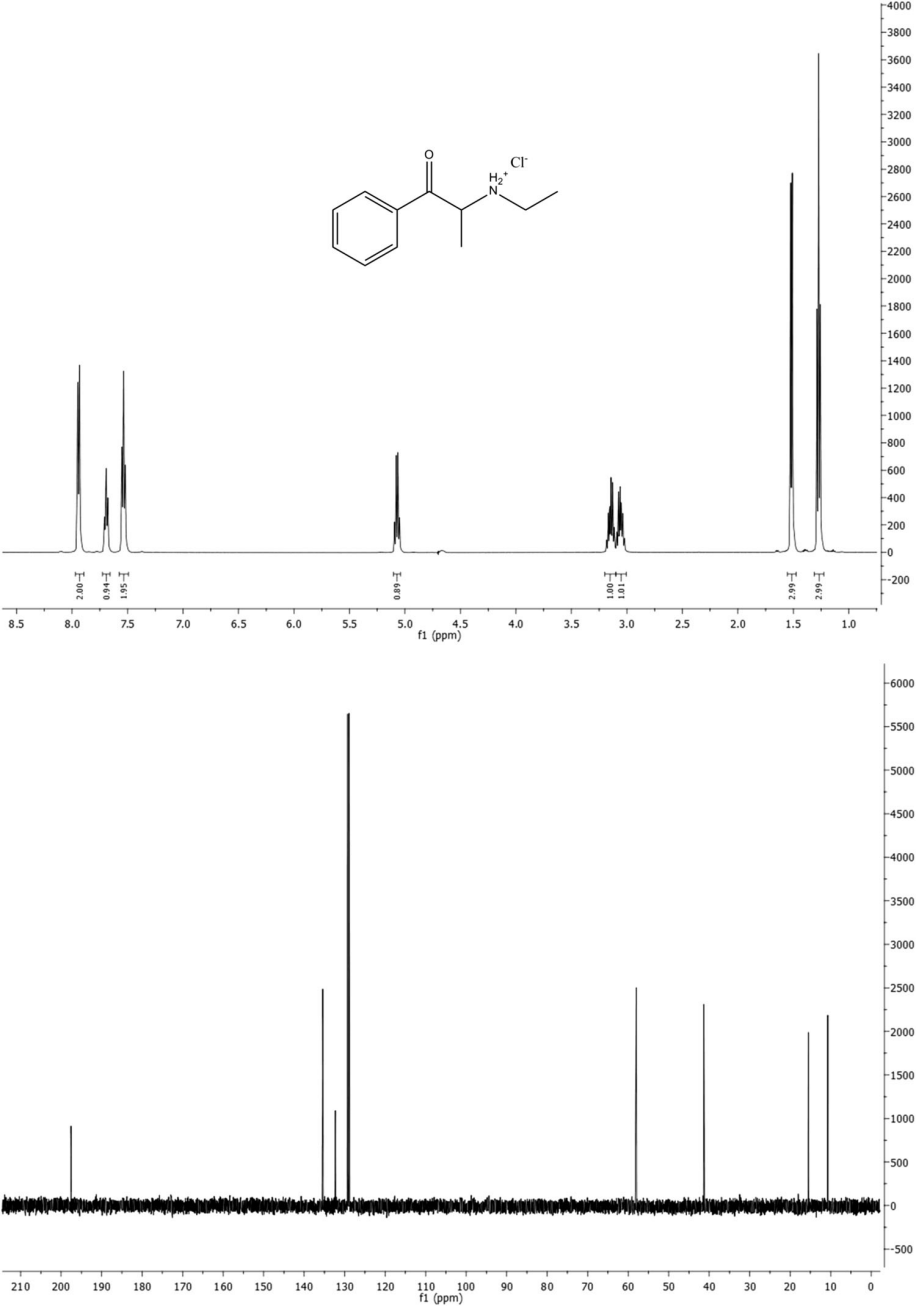
^1^H Nuclear magnetic resonance (NMR; *upper*) and ^13^C NMR (*lower*) spectra of compound **1** in D_2_O

**Fig. 3:**
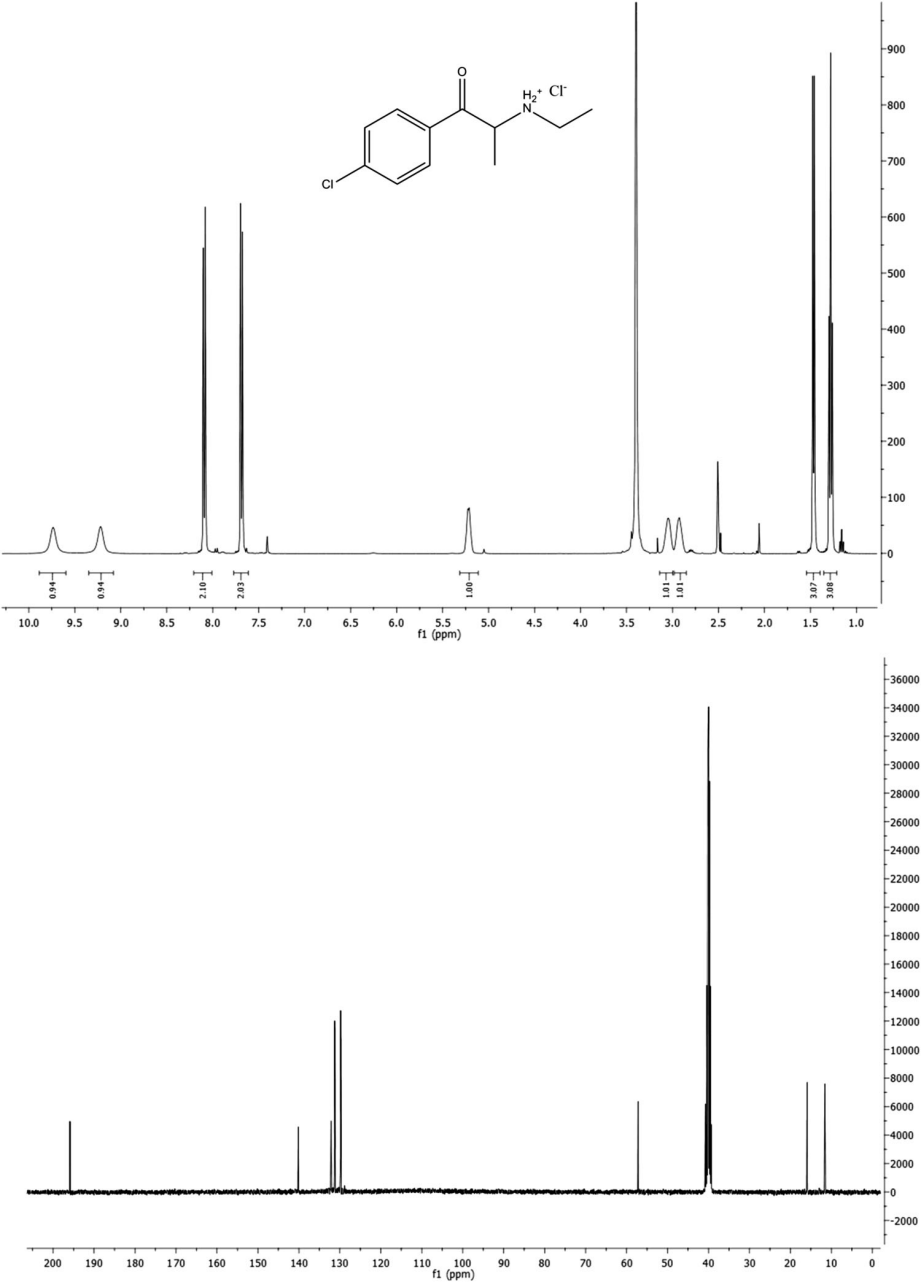
^1^H NMR (*upper*) and ^13^C NMR (*lower*) spectra of compound **2** in dimethyl sulfoxide-*d*_6_

Samples of both compounds received for analysis were examined by ^1^H and ^13^C NMR and IR spectroscopy, and ESI-MS, which confirmed that they were cathinone derivatives. Figures 2 and 3 show NMR spectra of compounds **1** and **2**, respectively. They fully confirm the predicted structures of the examined species. At the same time, they demonstrate that the examined substances are nearly pure cathinones. No other signals were detected which would imply the presence of other impurities in the examined sample of compound **1**. The ^1^H NMR spectrum of compound **2** showed signals at 7.95, 7.40, 5.05, 3.45, 3.16, 2.48, 2.06 and 1.18 ppm of very low intensities reflecting some undefined impurities (approximate content of impurities <1%; Fig. 3, upper panel). The N–H protons in the hydrochloride salt of compound **2** are diastereotopic (chemically inequivalent). These protons were observed as two broad singlets at δ = 9.74 and 9.22 ppm but only in DMSO-*d*_6_ solution (Fig. 3, upper panel). In D_2_O or CD_3_OD solutions, these signals were not observed (Fig. 2, upper panel). The ^1^H NMR spectra of both compounds confirmed the presence of a substituted benzene ring, which was mono-substituted (compound **1**) and di-substituted (*para,* compound **2**). The methinic protons in compounds **1** and **2** appeared as quintets at 5.07 and 5.21 ppm, respectively (see below). The *N*-ethyl side chains yielded two multiplets for methylene protons centered at 3.05 and 3.15 ppm for compound **1,** and two broad multiplets (like broad singlets) centered at 2.93 and 3.05 ppm for compound **2**. Terminal methyl groups yielded triplets centered at 1.27 and 1.28, for compounds **1** and **2**, respectively.

The ^13^C NMR spectra displayed carbonyl resonance at 197.6 and 195.9 ppm for compounds **1** and **2**, respectively (Figs. 2,3, lower panels, also see below). The six aromatic carbons appearing as four signals for both compounds showed greater differences because they reflect chemical and structural differences. The methylene carbons of *N*-ethyl groups were found at 41.3 and 40.8 ppm for compounds **1** and **2**, respectively. The methinic carbons resonated at 58.0 and 57.2 ppm for compounds **1** and **2**, respectively. The up-field two signals in both spectra correspond to both methyl carbons of each compound.

#### Compound **1** (ethcathinone hydrochloride)

^1^H NMR (D_2_O), δ (ppm): 7.94 (d, 2H), 7.69 (t, 1H), 7.53 (t, 2H), 5.07 (q, 1H), 3.15 (m, 1H), 3.05 (m, 1H), 1.52 (d, 3H), 1.27 (t, 3H).

^13^C NMR (D_2_O), δ (ppm): 197.6, 135.4, 132.3, 129.3, 128.9, 58.0, 41.3, 15.5, 10.7.

#### Compound **2** (4-CEC hydrochloride)

^1^H NMR (DMSO-*d*_6_), δ (ppm): 9.74 (bs, 1H), 9.22 (bs, 1H), 8.09 (d, 2H), 7.68 (d, 2H), 5.21 (q, 1H), 3.05 (bs, 1H), 2.93 (bs, 1H), 1.47 (d, 3H), 1.28 (t, 3H).

^13^C NMR (DMSO-*d*_6_), δ (ppm): 195.9, 140.1, 132.1, 131.2, 129.8, 57.2, 40.8, 15.9, 11.6.

### IR spectra

Only IR spectra of compound **1** were previously published [9]. The IR spectra showed strong C=O absorption bands at 1694 and 1686 cm^−1^ for compounds **1** and **2**, respectively. The shift of the C=O absorption band for one compound relative to the other is a result of a substituent in a *para* position of the aromatic ring in compound **2**. Spectra of both compounds showed characteristic bands associated with their salt forms. These included a broad pattern from 2300 to 3100 cm^−1^ corresponding to amine salt absorption bands and combination of aromatic and aliphatic C–H stretches. The main NH_2_^+^ stretch absorption for both compounds appeared at 2694 cm^−1^. The aromatic C=C ring stretch vibrations appeared at 1597 and ca. 1590 cm^−1^ for compounds **1** and **2**, respectively. A strong band at 839 cm^−1^ in the spectrum of compound **2** was characteristic for two adjacent free hydrogen atoms in an aromatic *para*-di-substituted ring (aromatic C–H out-of-plane bend). Two bands at 768 and 698 cm^−1^ in compound **1** were characteristic for an aromatic C–H out-of-plane bend of a mono-substituted benzene ring. All other absorption peaks are listed below.

#### Compound **1** (ethcathinone hydrochloride)

IR (KBr; cm^−1^): 3417, 2934, 2803, 2742, 2480, 1694, 1597, 1439, 1387, 1338, 1314, 1237, 1194, 1128, 1105, 1025, 978, 861, 792, 768, 698, 684, 657, 537.

#### Compound **2** (4-CEC hydrochloride)

IR (KBr; cm^−1^): 3530, 3430, 2983, 2795, 2694, 2447, 1687, 1590sh, 1554, 1462, 1392, 1292, 1234, 1164, 1094, 1051, 974, 919, 839, 794, 748, 684, 479.

### GC–MS and ESI-MS spectrometry

**Fig. 4:**
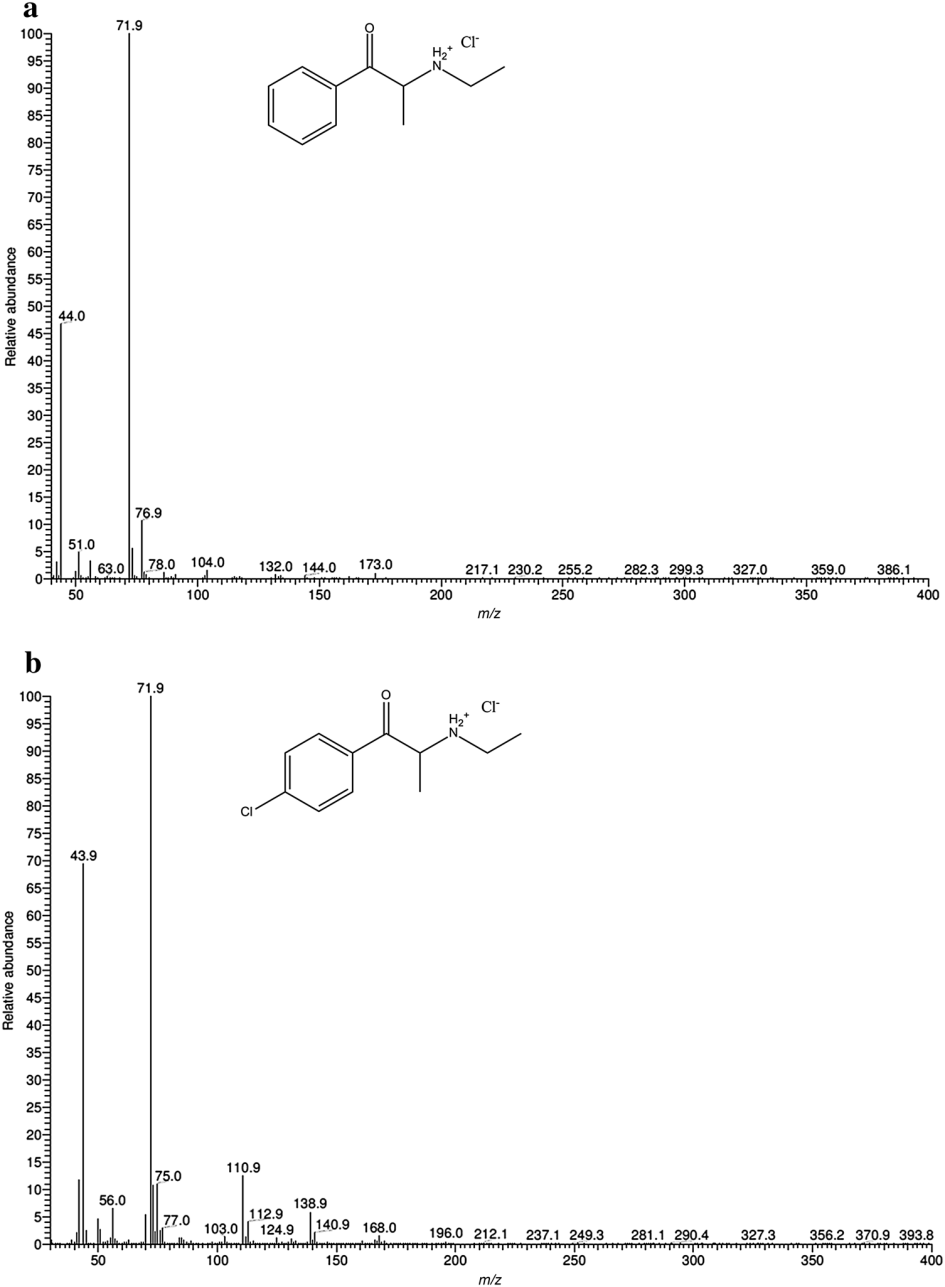
Electron ionization (EI) mass spectra of **a** compound **1** and **b** compound **2**

**Fig. 5:**
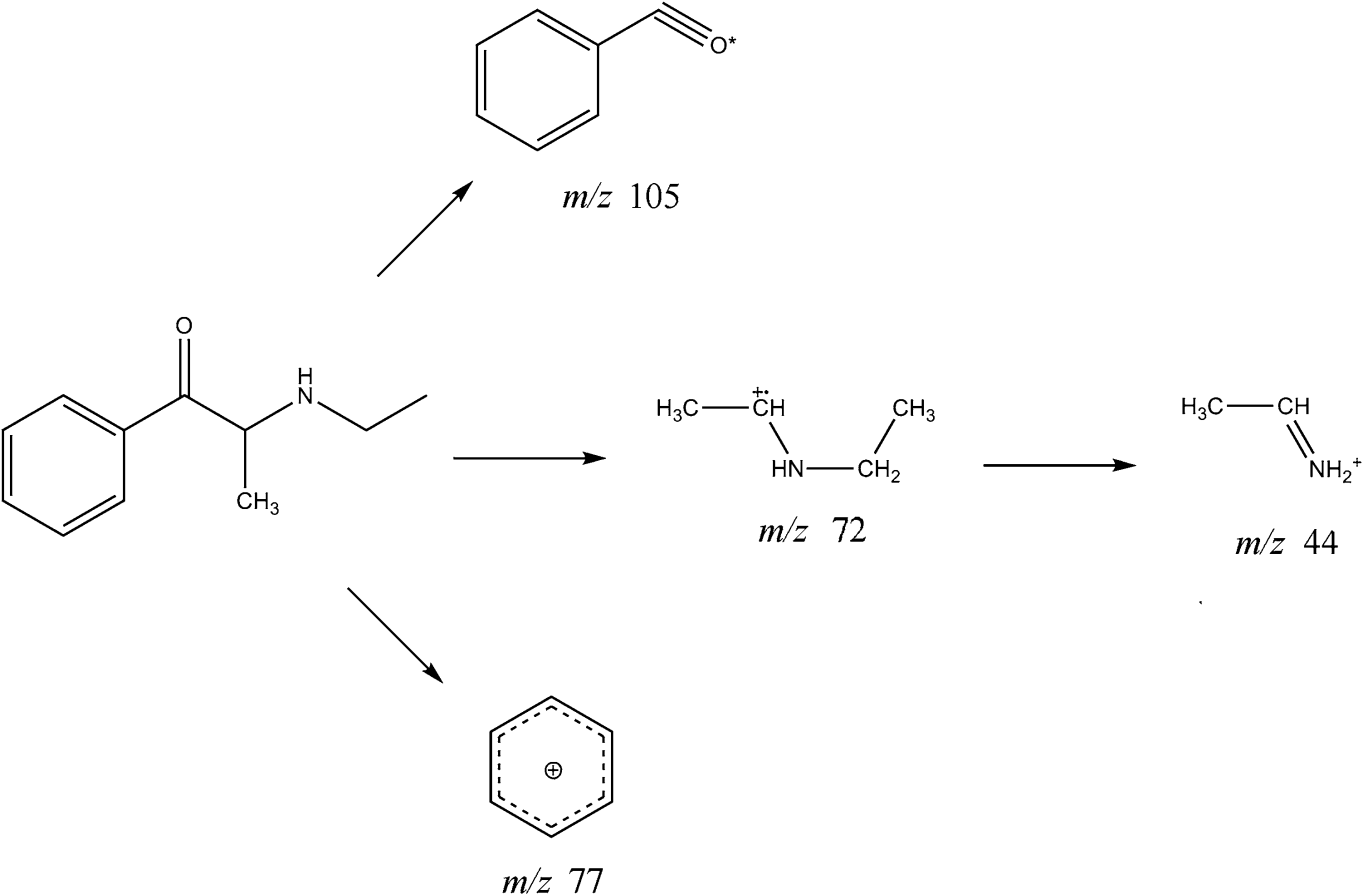
EI fragmentation pathways of compound **1**

Figure 4 shows the EI mass spectra of compounds **1** and **2**. Figure 5 shows EI fragmentation pathways of compound **1**. For compound **2**, fragmentation pathways was identical (with respect to the Cl substituent). The molecular ions for both compounds were not visible. Base peaks for compounds **1** and **2** at *m/z* 72 probably resulted from formation of *N*-ethylethano-1-iminium cation via the initiated benzoyl unit cleavage and by the loss of a hydrogen molecule. For compound **1**, after cleavage of the β-ketone unit, the benzoyl cation at *m/z* 105 was formed with subsequent loss of a CO molecule to form a phenyl cation at *m/z* 77. Similarly, for compound **2,** the same process led to the 4-chlorobenzoyl cation at *m/z* 139 and the next 4-chlorophenyl cation at *m/z* 111. These types of fragmentation are consistent with previously reported data [9, 17–19].

**Fig. 6:**
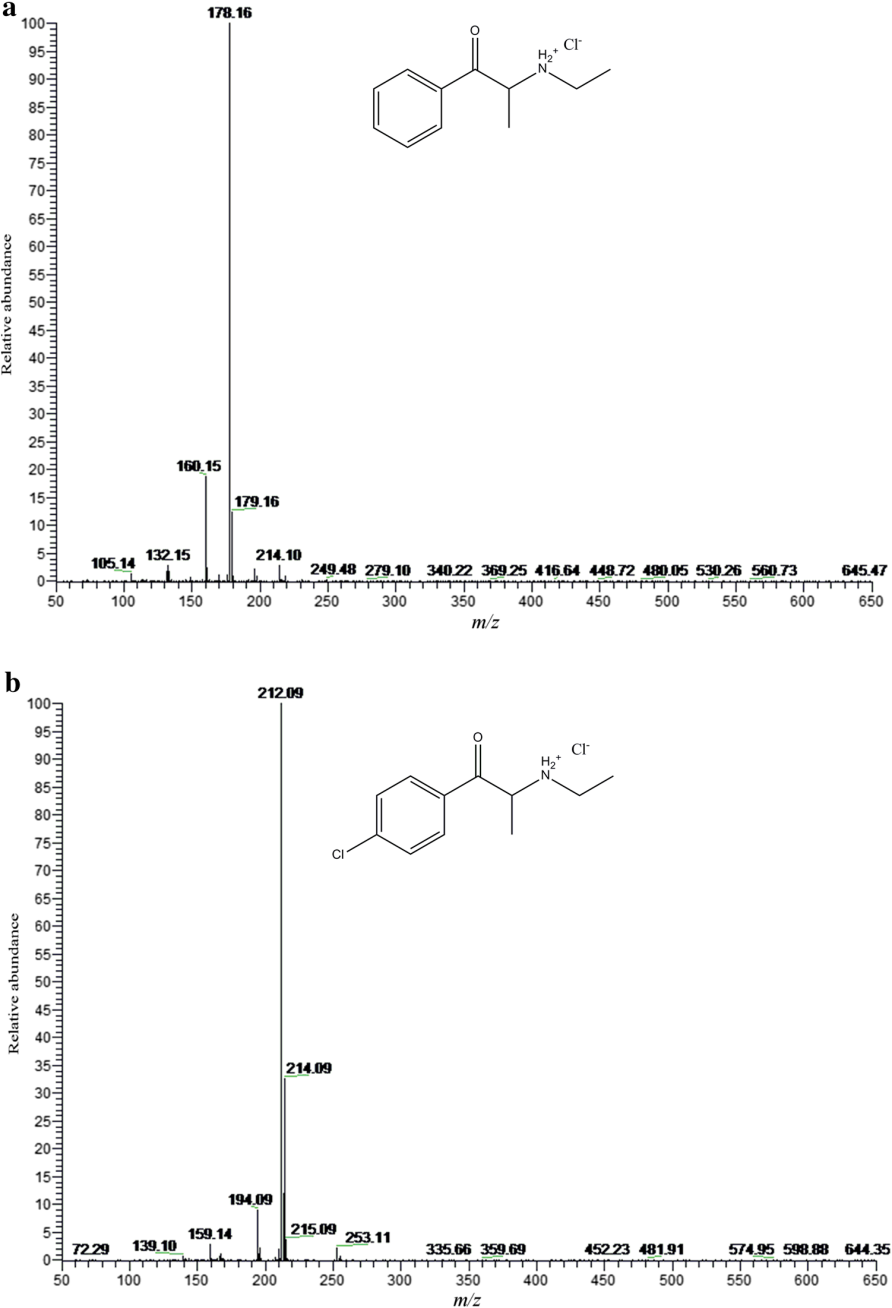
Electrospray ionization (ESI) mass spectra of **a** compound **1** and **b** compound **2**

Figure 6a shows the ESI mass spectrum of compound **1** upon HPLC–MS analysis. A base peak appeared at *m/z* 178.16 in good accordance with the calculated value for [M + H]^+^ [calc. for M (C_11_H_15_NO); *m/z* 178.12]. Figure 6b shows the ESI mass spectrum of compound **2**. Base peaks appeared at *m/z* 212.09 and 214.09 (for isotopic ^37^Cl atom). Both peaks were in good accordance with calculated values for [M + H]^+^ ions [calc. for M(C_11_H_14_ClNO); *m/z* 212.08 and 214.08 for ^35^Cl and ^37^Cl, respectively]. Isomer ratio occurring in nature was ca. 76/34 (^35^Cl/^37^Cl) which is almost ideally reflected by the intensities of both basic peaks.

**Fig. 7:**
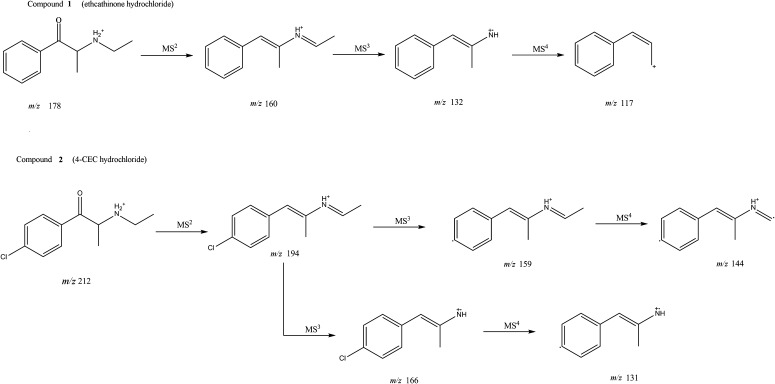
Proposed fragmentation pathways of compounds **1** and **2** in ESI-MS^n^ modes

The elimination of one water molecule from the protonated molecular ion is very characteristic for cathinones, especially in the tandem mode [19]. For both described compounds, this phenomenon appeared in MS^1^ spectra. In Fig. 7, we present proposed fragmentation pathways for ESI in MS^2^, MS^3^ and MS^4^ modes for compound **1** and compound **2** as well.

### X-ray studies

**Fig. 8:**
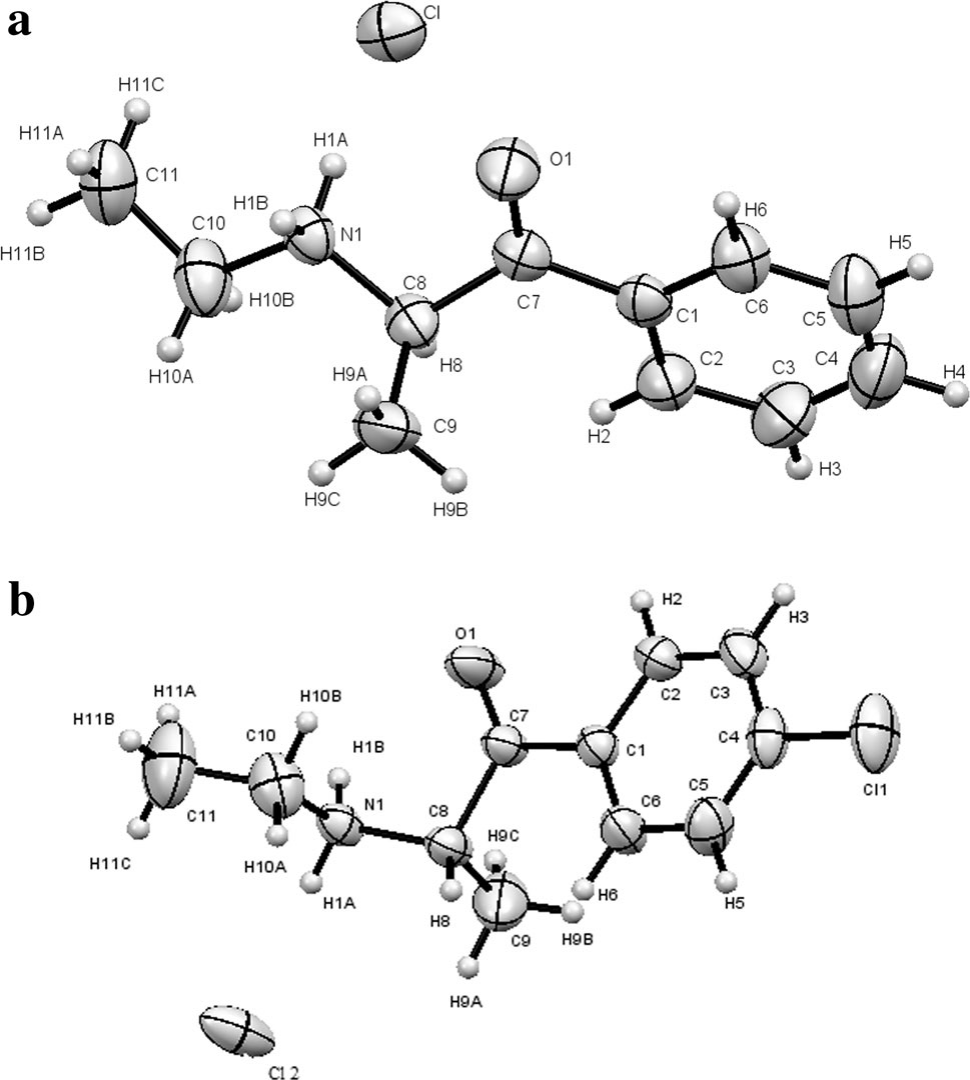
**a** The molecule of an (*S*) enantiomer of compound **1** in the crystal. **b** The molecule of an (*R*) enantiomer of compound **2** in the crystal. Ellipsoids correspond to 50% probability levels

**Fig. 9:**
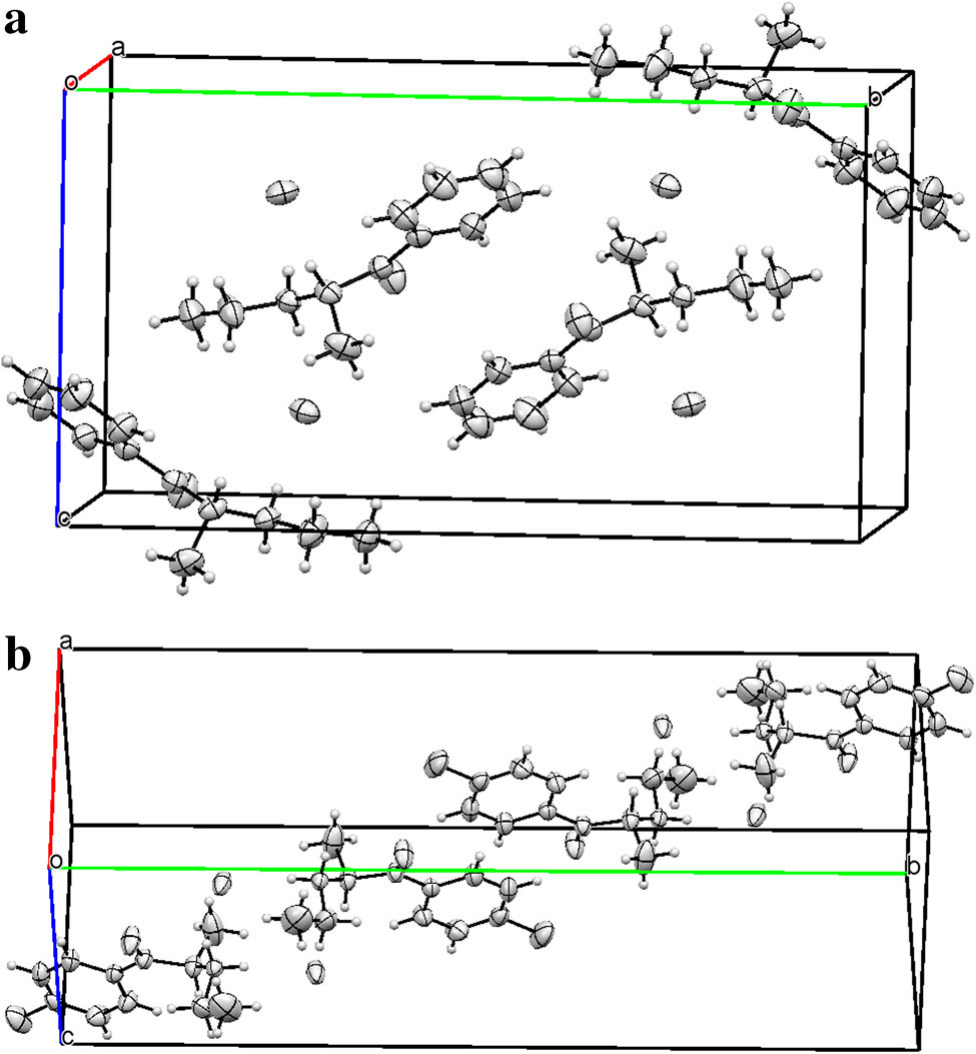
Packing diagrams of **a** compound **1** and **b** compound **2**

Both hydrochloride salts crystallize in a monoclinic (P2_1_/c) space group. The molecular structures of both compounds are shown in Fig. 8. Packing diagrams for both compounds are shown in Fig. 9. Crystal data and structure refinement for compounds **1** and **2** are presented in Table 1. The ring systems in both compounds were planar. All distances and angles in the molecular structures were normal. Hydrogen bonds in compounds **1** and **2** are listed in Tables 2 and 3, respectively. Selected geometric parameters for compounds **1** and **2** are presented in Table 4.

**Table 1:** Crystal data and structure refinement for ethcathinone hydrochloride (compound **1**) and 4-CEC hydrochloride (compound **2**)

	Compound **1**	Compound **2**
Molecular formula	C_11_H_16_NOCl	C_11_H_15_NOCl_2_
Molecular weight	213.70	248.14
Crystal system	Monoclinic	Monoclinic
Space group	P2_1_/c	P2_1_/c
Temperature (K)	290	290
a (Å)	5.6558(1)	7.3569(3)
b (Å)	19.7808(6)	26.1246(5)
c (Å)	10.5559(2)	7.2462(2)
β (°)	97.570(2)	112.454(4)
V (Å^3^)	1170.66(5)	1287.11(8)
Z	4	4
Dx (g cm^−3^)	1.212	1.281
Absorption coeff. (mm^−1^)	2.64	0.48
F (000)	456	520
Crystal size (mm)	0.13 × 0.07 × 0.02	0.60 × 0.24 × 0.20
Data collection and structure solution		
Data collected	12,532	18,874
Independent reflections	2381	2615
Observed reflections [I > 2σ(I)]	2065	2407
R(int.)	0.021	0.018
Completeness (%)	99.8	99.5
*T*_max_/*T*_min_	1.000/0.838	1.000/0.858
No of parameters	154	138
R1[I > 2σ(I)]	0.0323	0.0394
*wR2* (all data)	0.0918	0.0976
*S*	1.04	1.05
Largest difference peak and hole (eÅ^−3^)	0.25, −0.14	0.37, −0.47

**Table 2:** Hydrogen bonds in compound **1** (Å and deg)

D–H···A	D–H	H···A	D···A	D–H···A
N1-H1*A*···Cl1	0.883(19)	2.25(2)	3.1307(13)	174.4(15)
N1-H1*B*···Cl1^i^	0.887(18)	2.299(19)	3.1692(13)	167.0(14)
C8-H8···Cl1^ii^	0.957(16)	2.919(16)	3.8234(14)	158.0(13)
C9-H9*B*···O1^ii^	0.95(2)	2.54(2)	3.183(2)	125.2(17)
C9-H9*C*···Cl1^iii^	0.96(2)	2.88(2)	3.7320(18)	148.3(18)
C11-H11*A*···Cl1^i^	0.99(3)	2.95(3)	3.712(2)	134.6(18)

Symmetry codes: (i) *x*, −*y* + 3/2, *z* − 1/2; (ii) *x* + 1, *y*, *z*; (iii) *x* + 1, −*y* + 3/2, *z* − 1/2

**Table 3:** Hydrogen bonds in compound **2** (Å and deg)

D–H···A	D–H	H···A	D···A	D–H···A
N1-H1*A*···Cl2	0.91(2)	2.20(2)	3.1064(15)	174.3(17)
N1-H1*B*···Cl2^i^	0.88(2)	2.31(2)	3.1372(15)	157.3(18)
C8-H8···Cl2^ii^	0.94(2)	2.62(2)	3.5488(18)	167.6(17)
C10-H10*B*···Cl1^iii^	0.98(3)	2.94(3)	3.610(2)	126.5(17)

Symmetry codes: (i) *x*, −*y* + 1/2, *z* − 1/2; (ii) *x*, −*y* + 1/2, *z* + 1/2; (iii) −*x*, −*y* + 1, −*z* + 1

**Table 4:** Selected geometric parameters for compounds **1** and **2** (*Å*, ^o^)

	Compound **1**	Compound **2**
O1-C7	1.2080(16)	1.210(2)
N1-C10	1.4917(19)	1.488(2)
N1-C8	1.4921(18)	1.485(2)
C7-C8	1.5143(19)	1.522(2)
C8-C9	1.523(2)	1.514(3)
C10-C11	1.497(3)	1.481(3)
O1-C7-C1	121.34(12)	121.99(15)
O1-C7-C8	119.14(12)	119.10(15)
C1-C7-C8	119.44(11)	118.87(13)
N1-C8-C7	107.94(11)	109.47(13)
N1-C8-C9	111.03(12)	109.22(15)
C7-C8-C9	108.46(13)	109.34(16)
N1-C10-C11	110.72(15)	111.50(18)
C2-C1-C7-O1	−165.45(14)	−8.7(3)
C6-C1-C7-O1	12.8(2)	168.50(18)
C2-C1-C7-C8	17.7(2)	168.87(16)
C6-C1-C7-C8	−164.05(13)	−13.9(3)
C10-N1-C8-C7	165.77(13)	−69.25(19)
C10-N1-C8-C9	−75.47(16)	171.06(18)
O1-C7-C8-N1	25.19(18)	−26.5(2)
C1-C7-C8-N1	−157.87(11)	155.85(14)
O1-C7-C8-C9	−95.20(16)	93.1(2)
C1-C7-C8-C9	81.74(15)	−84.5(2)
C8-N1-C10-C11	−174.37(15)	−179.8(2)

#### Compound **1**

**Fig. 10:**
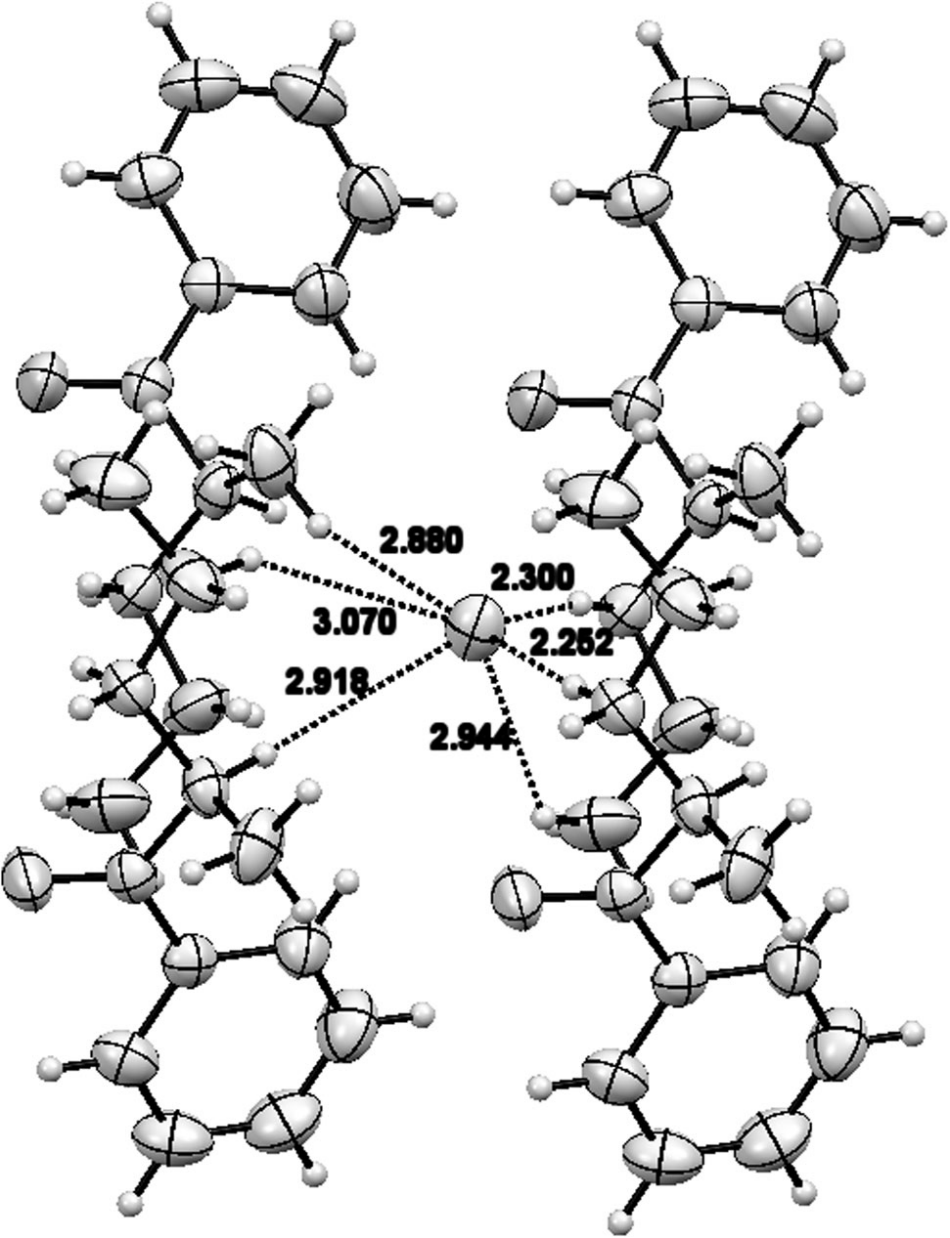
Schematic representation of a single chloride ion surrounded by compound **1** molecules. The *dotted lines* show short contacts of the chloride ion with hydrogen atoms from four nearest molecules

Both compounds occurred in the examined crystals as paired enantiomers. The pairs were mutually linked by hydrogen bonds formed with chloride ions occurring between these molecules. For compound **1**, all short contacts were linked to methyl groups (in position 2, and in ethyl group) and chloride ions freely located between molecules of the compound as well as with a ketone group of oxygen atoms. Chloride ions were placed at almost equal distance from two ammonium groups from the adjacent molecules (3.131 and 3.169 Å, respectively, Fig. 9a); at the same time, hydrogen atoms C–H9 and C–H8 from two surrounding molecules formed close contacts with the same chloride ion (2.88 and 2.918 Å, respectively, Fig. 10.). Torsional angles C7C8N1C10 were identical in both enantiomers present in the elementary cell (165.77^o^).

#### Compound **2**

**Fig. 11:**
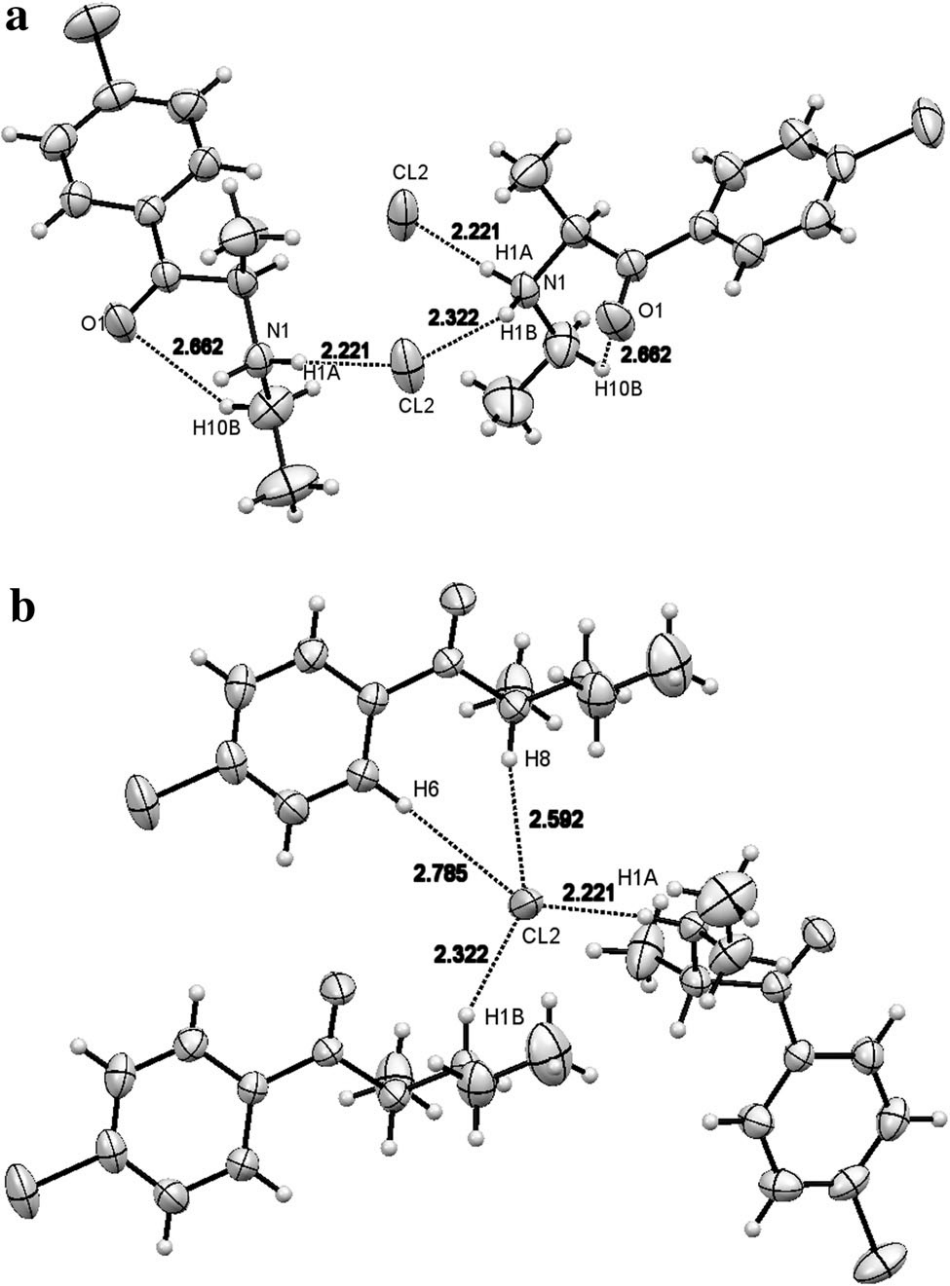
**a** Two molecules forming half an elementary cell of compound **2** with short interatomic distances are shown. **b** Three molecules of compound **2** surrounding the chloride anion in the crystallographic structure. The *dotted lines* show short contacts of chloride ion with hydrogen atoms from three adjacent molecules

Compound **2** stereoisomers occurred pairwise forming hydrogen bonds with chloride ions (Fig. 11). Torsional angles C7C8N1C10 are identical in both enantiomers (69.25°). Distances between N-HA, as well as N-HB, and chloride ion present between these molecules were 2.221 and 2.322Å, respectively.

Crystallographic structures of both compounds can be characterized by the distance between the chloride ion and the surrounding molecules of cathinone cation. In the case of compound **1**, the chloride ion was surrounded by four different cationic species (Fig. 10), whereas in compound **2**, this ion was surrounded by three adjacent cations (Fig. 11b). This finding is corroborated by short distances between the chloride ion and various fragments of surrounding molecules (<3Å).

## Conclusions

In this paper, we report spectroscopic (IR, NMR and MS) and crystallographic characteristics of two designer drugs: ethcathinone and 4-chloroethcathinone (4-CEC), both present on the illicit drug market either as pure substance or mixed with other species (to be confirmed chromatographically as well as by using NMR and MS). The examined samples of these two compounds proved, indeed, to be either very pure (compound **1**) or mixed with other substances (compound **2**). NMR and MS spectra can be used to differentiate between both compounds. Crystallographic studies confirmed the occurrence of both compounds as racemic mixtures which would rather be expected considering the possible method of synthesizing these compounds.
